# Proof-of-concept study: profile of circulating microRNAs in Bovine serum harvested during acute and persistent FMDV infection

**DOI:** 10.1186/s12985-017-0743-3

**Published:** 2017-04-07

**Authors:** Carolina Stenfeldt, Jonathan Arzt, George Smoliga, Michael LaRocco, Joseph Gutkoska, Paul Lawrence

**Affiliations:** Plum Island Animal Disease Center, USDA/ARS/NAA/FADRU, P.O. Box 848, Greenport, NY 11944-0848 USA

**Keywords:** microRNA (miRNA), Foot-and-mouth disease virus (FMDV), Bovine miRNA profile, Serum, Persistence

## Abstract

**Background:**

Changes in the levels of circulating microRNAs (miRNAs) in the serum of humans and animals have been detected as a result of infection with a variety of viruses. However, to date, such a miRNA profiling study has not been conducted for foot-and-mouth disease virus (FMDV) infection.

**Methods:**

The relative abundance of 169 miRNAs was measured in bovine serum collected at three different phases of FMDV infection in a proof-of-concept study using miRNA PCR array plates.

**Results:**

Alterations in specific miRNA levels were detected in serum during acute, persistent, and convalescent phases of FMDV infection. Subclinical FMDV persistence produced a circulating miRNA profile distinct from cattle that had cleared infection. bta-miR-17-5p was highest expressed during acute infection, whereas bta-miR-31 was the highest during FMDV persistence. Interestingly, miR-1281was significantly down-regulated during both acute and persistent infection. Cattle that cleared infection resembled the baseline profile, adding support to applying serum miRNA profiling for identification of sub-clinically infected FMDV carriers. Significantly regulated miRNAs during acute or persistent infection were associated with cellular proliferation, apoptosis, modulation of the immune response, and lipid metabolism.

**Conclusions:**

These findings suggest a role for non-coding regulatory RNAs in FMDV infection of cattle. Future studies will delineate the individual contributions of the reported miRNAs to FMDV replication, determine if this miRNA signature is applicable across all FMDV serotypes, and may facilitate development of novel diagnostic applications.

**Electronic supplementary material:**

The online version of this article (doi:10.1186/s12985-017-0743-3) contains supplementary material, which is available to authorized users.

## Background

Foot-and-mouth disease virus (FMDV) is the etiological agent for foot-and-mouth disease (FMD); one of the most contagious animal diseases known. FMD targets both domestic and wild cloven-hoofed animals, and outbreaks affecting important livestock populations, such as cattle, sheep, and pigs incur enormous economic, political, and social ramifications. Traditional containment strategies combine mass vaccination with large scale animal culling. FMDV is represented globally by seven different serotypes: O, A, C, Asia1, SAT1, SAT2, and SAT3; and within each serotype there are multiple subtypes and variants. The virus is the prototypic member of the Aphthovirus genus of the family *Picornaviridae* and possesses a single-stranded positive-sense RNA genome of approximately 8500 nucleotides (nts), which consists of a large open reading frame (ORF) flanked by 5’ and 3’ non-translated regions (NTRs). The positive-sense RNA functions as a messenger RNA (mRNA) from which a single polyprotein is translated and subsequently proteolyzed into a series of intermediate and mature viral proteins. The ORF is traditionally sub-divided into three regions: P1, P2, and P3. The P1 region encodes four structural proteins, which assemble into the FMDV capsid, whereas the P2 and P3 regions encode the virus non-structural proteins essential for virus replication. The 5’ and 3’ NTRs flanking the ORF exhibit multiple secondary RNA structures, which regulate the stepwise translation of virus proteins and replication of the RNA genome. Knowledge obtained from the study of the molecular biology and pathogenesis of FMDV continues to be applied to the development of improved vaccines and anti-viral therapies [1, 2].

RNA interference (RNAi), a process by which mRNA transcripts are targeted for degradation or translational repression by short non-coding RNA molecules (ncRNAs) has been widely studied for its utility as an anti-viral therapeutic platform. Small interfering RNAs (siRNAs) are generated when large double-stranded RNA (dsRNA) molecules are gradually processed into progressively smaller dsRNA of approximately 20–22 nucleotides in length; and have been investigated as anti-viral therapeutics [3–5]. In the case of FMDV, siRNAs targeted to sequences in the virus genome have been tested for their capacity to impede the progression of viral infection with reportedly mixed benefits [6–9]. A key drawback of siRNA-based anti-viral therapies is the required complete sequence complementarity between the siRNA and its cognate RNA target. RNA viruses are highly mutable, and as such, the accumulation of mutations with each replication cycle diminishes the efficacy of the siRNA treatment. Additionally, with seven different serotypes and multiple subtypes and variants of FMDV, designing siRNAs that are universally functional across virus strains would be highly challenging.

In contrast to siRNAs, microRNAs (miRNAs) do not require perfect complementarity to their target RNA molecule [10–13], which mitigates the effect of mutations in the RNA genome and facilitates a similar function across multiple forms of FMDV. Another key difference between siRNAs and miRNAs is that siRNAs are generally produced from exogenous RNA molecules, while miRNAs are generated from endogenous RNA molecules [12]. In the normal cellular environment, miRNAs act as key post-transcriptional regulators of gene expression for a variety of biological processes including immune response, cell proliferation, and metabolism [14–16].

Currently, miRNAs are also being explored as potential bio-markers of infectious disease, particularly miRNAs that are detectable in the bloodstream [17–21]. Studies have shown that different viruses generate distinct expression profiles of circulating miRNA, so much so that one group was able to distinguish between enterovirus 71 and coxsackievirus 16 as the etiological agents for individual cases of hand-foot-and-mouth disease [17]. Unfortunately, while greater than 2588 human miRNAs have been identified, only approximately 798 bovine miRNAs have been fully characterized (release 21, June 2014, miRBase.org). Furthermore, while the miRNA response to several bacterial pathogens has been examined in cattle, very few studies have been conducted in the context of viral infection [22].

Although still an evolving technology, the information gained from miRNA profiling investigations has contributed substantially to efforts for curtailing viral infection. One strategy has been to incorporate miRNA target sequences into viral genomes that target host anti-viral response pathways to contribute to the development of live attenuated vaccine platforms, which has been accomplished for vesicular stomatitis virus [23], Japanese encephalitis virus [24], coxsackievirus A21 [23], and influenza virus [25, 26]. Contrastingly, some viruses have exploited the opposite approach, as several DNA viruses and a few RNA viruses have been identified that harbor functional miRNA sequences [27–29]. Of relevance to FMDV, miRNA sequences were identified in the anti-genome (or negative strand intermediate) of a representative Picornavirus, hepatitis A virus (HAV), thus demonstrating that RNA viruses, even those whose replication cycle is confined to the cytoplasm, can encode functional miRNAs like their DNA virus counterparts [28, 29]. Although certainly still controversial, these findings contest the notion that RNA viruses would not encode functional miRNAs since interactions with the cellular miRNA machinery would require: (i) localization of an RNA virus genome to the nucleus and (ii) that processing of the pre-miRNA hairpin would irreparably damage the virus genome.

An alternative anti-viral miRNA strategy has been to introduce artificial miRNAs in cell culture or in infected animals to target specific viral sequences or the mRNA transcripts of cellular proteins that the virus requires for an efficient replication cycle. This approach has been beneficially used in two reported instances involving FMDV, where artificial miRNAs were constructed that targeted either the IRES sequence in the 5’ NTR or the coding sequence for the 3D polymerase [30, 31]. When these constructs were transfected into cell culture prior to FMDV infection, replication was significantly impeded. In another study, the artificial miRNA sequences were inserted into the genomic DNA of a cell line, so as to allow for constitutive expression of the anti-FMDV miRNAs [32]. However, this failed to protect the cells from FMDV infection due to inefficient localization of the anti-FMDV miRNAs to sites of virus replication in the sub-cellular environment.

**Fig. 1:**
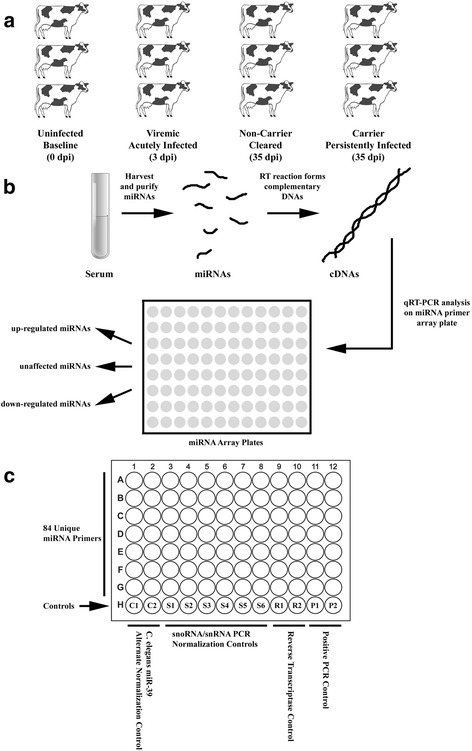
Schematic overview of miRNA profiling study. **a** Serum was collected from three different groups of FMDV-infected cattle: acutely infected (viremic; 3–4 dpi), persistently infected (“FMDV carriers”; 35 dpi) and convalescent (“non-carriers”; 35 dpi), and were compared to uninfected controls. Each group comprised serum samples from three animals. **b** miRNAs were purified from individual serum samples. The purified miRNAs were reverse transcribed into complementary DNA (cDNA). The cDNA samples were then analyzed by RT-PCR on bovine miRNome miRNA array plates containing primers to 169 different bovine miRNAs. The results obtained indicated which miRNAs were left unchanged, up-regulated, or down-regulated in response to FMDV infection. **c** schematic of the organization of the miRNA PCR array plates utilized in this study

This current work comprises a proof-of-concept study in which the differential regulation of serum miRNAs was investigated in cattle infected with FMDV serotype A isolate A24 Cruzeiro. The bovine serum miRNA profile was characterized using quantitative reverse transcriptase polymerase chain reaction (RT-qPCR) array plates designed to detect the relative abundance levels of 169 of the bovine miRNAs characterized in the past several years (Table 1, Fig. 1c). Serum from uninfected animals was compared to samples obtained through three distinct phases of FMDV infection: acute infection (peak viremia), persistent infection (subclinical persistence of FMDV in the upper respiratory tract), and convalescent phase, comprising animals that had successfully cleared the infection (Fig. 1a, b). Distinct miRNA signatures of up- and down-regulated miRNAs in circulation in response to FMDV infection were detected. The results obtained from this proof-of-concept study reinforce the application of this approach to future large scale testing and the investigation of miRNA signatures for other serotypes of FMDV.

**Table 1:** Bovine miRNA array

miRNA					
bta-miR-1	bta-miR-30a-5p	bta-miR-127	bta-miR-150	bta-miR-222	bta-miR-1179
bta-miR-7	bta-miR-30b-5p	bta-miR-128	bta-miR-151-3p	bta-miR-320a	bta-miR-1185
bta-miR-10a	bta-miR-30c	bta-miR-129-3p	bta-miR-151-5p	bta-miR-331-3p	bta-miR-1193
bta-miR-10b	bta-miR-30d	bta-miR-129-5p	bta-miR-152	bta-miR-342	bta-miR-1197
bta-miR-15a	bta-miR-30e-5p	bta-miR-130a	bta-miR-153	bta-miR-345-5p	bta-miR-1224
bta-miR-15b	bta-miR-31	bta-miR-130b	bta-miR-154a	bta-miR-361	bta-miR-1225-3p
bta-miR-16b	bta-miR-34a	bta-miR-132	bta-miR-181a	bta-miR-363	bta-miR-1248
bta-miR-17-3p	bta-miR-34b	bta-miR-133a	bta-miR-181b	bta-miR-365-3p	bta-miR-1249
bta-miR-17-5p	bta-miR-34c	bta-miR-133b	bta-miR-181c	bta-miR-369-3p	bta-miR-1256
bta-miR-18a	bta-miR-93	bta-miR-134	bta-miR-186	bta-miR-369-5p	bta-miR-1271
bta-miR-18b	bta-miR-98	bta-miR-135a	bta-miR-191	bta-miR-374a	bta-miR-1281
bta-miR-19a	bta-miR-99a-5p	bta-miR-135b	bta-miR-192	bta-miR-380-3p	bta-miR-1282
bta-miR-19b	bta-miR-99b	bta-miR-136	bta-miR-193a-3p	bta-miR-380-5p	bta-miR-1287
bta-miR-20a	bta-miR-100	bta-miR-137	bta-miR-193a-5p	bta-miR-423-3p	bta-miR-1291
bta-miR-20b	bta-miR-101	bta-miR-138	bta-miR-195	bta-miR-425-3p	bta-miR-1296
bta-miR-21-3p	bta-miR-103	bta-miR-139	bta-miR-199a-3p	bta-miR-425-5p	bta-miR-1298
bta-miR-21-5p	bta-miR-105a	bta-miR-140	bta-miR-199a-5p	bta-miR-450a	bta-miR-1301
bta-miR-22-5p	bta-miR-105b	bta-miR-141	bta-miR-199b	bta-miR-455-3p	bta-miR-1306
bta-miR-23a	bta-miR-106a	bta-miR-142-3p	bta-miR-200a	bta-miR-455-5p	bta-miR-1307
bta-miR-23b-3p	bta-miR-106b	bta-miR-142-5p	bta-miR-200b	bta-miR-484	bta-let-7a-3p
bta-miR-24-3p	bta-miR-107	bta-miR-143	bta-miR-200c	bta-miR-487a	bta-let-7a-5p
bta-miR-25	bta-miR-122	bta-miR-144	bta-miR-204	bta-miR-487b	bta-let-7b
bta-miR-26a	bta-miR-124a	bta-miR-145	bta-miR-205	bta-miR-497	bta-let-7c
bta-miR-26b	bta-miR-124b	bta-miR-146a	bta-miR-210	bta-miR-499	bta-let-7d
bta-miR-27a-3p	bta-miR-125a	bta-miR-146b	bta-miR-214	bta-miR-532	bta-let-7e
bta-miR-27b	bta-miR-125b	bta-miR-147	bta-miR-215	bta-miR-545-3p	bta-let-7f
bta-miR-29a	bta-miR-126-3p	bta-miR-148a	bta-miR-218	bta-miR-545-5p	bta-let-7 g
bta-miR-29b	bta-miR-126-5p	bta-miR-148b	bta-miR-221	bta-miR-660	bta-let-7i
bta-miR-29c					

Panel of 169 established bovine miRNAs that were profiled for differential expression in response to FMDV infection in cattle serum

## Methods

### Animals

Serum samples used for this investigation were derived from non-vaccinated animals included in studies investigating the pathogenesis of acute and persistent phases of FMDV infection in cattle [33, 34]. The cattle used were ~200 kg (kg) Holstein cattle that were 6–8 months old, and were purchased from Thomas D. Morris Inc. (Reisterstown, MD). Briefly, cattle were infected with FMDV serotype A isolate A24 Cruzeiro via intra-nasopharyngeal (INP) inoculation [35] at a dose of 10^5^ BTID_50_ (50% infectious doses titrated using bovine tongue). The INP inoculation method represents a needle-free “simulated-natural system”, where a volume of inoculum is introduced to the nasopharynx of cattle via a plastic catheter that has been calibrated for optimal deposition. The procedures employed for this study were approved by the Plum Island Animal Disease Center (PIADC) Institutional Animal Care and Use Committee (IACUC) under protocols: 209-12-R and 209-15-R.

### Serum sample collection

Whole blood was collected in serum separation tubes, which were stored on ice until downstream centrifugation in the laboratory for harvesting the serum. The serum was then stored at −70 ° C until ready for subsequent laboratory testing. Serum samples for miRNA profiling came from whole blood harvested during acute infection, which was defined as the day on which peak viremia occurred (3 or 4 days post-inoculation [dpi]; data not shown). Additional samples were obtained during the persistent phase of infection (~35 dpi) from cattle that were defined as either persistently infected “FMDV carriers”, or from animals that had successfully cleared the infection; “non-carriers”/convalescent animals. The distinction between FMDV carriers and non-carriers was made based on detection of FMDV in oropharyngeal fluids beyond 28 dpi (data not shown) [34]. Serum collected from cattle prior to inoculation with FMDV was used as a baseline for comparison with infected and convalescent animals. Each test group comprised sera from three individual animals. Samples used in this study are listed in Additional file 1: Table S3.

### miRNA isolation

miRNAs were harvested from serum samples using the miRNeasy serum/plasma kit (Qiagen, catalog # 217184) following manufacturer’s instructions. The miRNA harvesting process included the addition of the miRNeasy serum/plasma spike-in control (Qiagen, catalog # 219610) to facilitate downstream quality control. Extracted miRNA was immediately used for cDNA synthesis.

### cDNA synthesis

cDNA was generated from purified miRNA using the miScript II RT (reverse transcriptase) kit purchased from Qiagen (Catalog # 218160) following manufacturer’s instructions. Reaction mixes were incubated at 37 ° C for 60 min, followed by 5 min at 95 ° C, and finally cooled to 4 ° C. The cDNA samples were then stored at −70 ° C until subsequent analysis.

### miScript miRNA PCR array cow miRNome™ profiler plates

Ninety-six well miRNA PCR array plates for the bovine miRNome™ were purchased from Qiagen (Catalog # 331221, Product # MIBT-651Z and MIBT-652Z). In addition to wells containing primers for the miRNA species listed in Table 1 and shown in Fig. 1, both array plates possessed wells containing *C. elegans* miR-39, snoRNA, and snRNA for normalization of the data as well as a reverse transcription control (miRTC) and positive control for the PCR reaction (PPC).

### Reverse transcription quantitative PCR (RT-qPCR)

The cDNA generated in the prior RT step was used as template material for the downstream RT-qPCR analysis. The cDNA template was diluted and mixed with the reagents provided in the miScript SYBR Green PCR kit purchased from Qiagen (Catalog # 218076), consisting of QuantiTect SYBR green master mix and miScript universal primer. 25 μL of combined template and reagents was loaded onto Qiagen bovine miRnome™ miScript miRNA PCR array plates 1 and 2 (Product # MIBT-651Z and MIBT-652Z). The PCR array plates were analyzed on ABI 7500 thermal cyclers (Applied Biosystems, Life Technologies, Foster City, CA) using the following cycling parameters: an initial activation of the HotStar™ Taq DNA polymerase at 95 ° C for 15 min, followed by 40 iterative cycles of 15 s (sec) at 94 ° C, 30 s at 55 ° C, and 30 s at 70 ° C.

### miRNA expression profile analysis

The C_t_-values accumulated from the RT-PCR analyses were analyzed using the Qiagen GeneGlobe (http://www.qiagen.com/us/shop/genes-and-pathways/data-analysis-center-overview-page/mirna-pcr-array-mirbase-profiler-plates-data-analysis-center/) online software platform (formerly SABiosciences: pcrdataanalysis.sabiosciences.com/mirna/arrayanalysis.php). The quality of the miRNA extractions, RT reactions and PCR efficiency were evaluated against internal controls included on each PCR array plate. The data was normalized using the “global C_t_ mean of expressed miRNAs” method provided in the data analysis platform. Post-normalization of the C_t_ values, the fold changes in miRNA expression in each infected and convalescent sample relative to control samples was calculated using the ∆∆C_t_ method [36]. The significance of the fold-changes was evaluated using two-tailed t-tests comparing each test group (acute, FMDV carrier, and non-carrier) to the uninfected control group. Significant levels were set at fold change >1.50 and *p*-value < 0.05.

### Cell culture

LFBK-αvβ6 cells [37, 38] were generously provided by Dr. Luis Rodriguez (PIADC, ARS, USDA). The cells were cultured in Dulbecco’s minimal essential medium (DMEM) supplemented with 10% fetal bovine serum (FBS) and 1% antibiotic/antimycotic (A/A) at 37 ° C in humidified incubator with 5% CO_2_.

### miRNA mimic transfection and infection

LFBK-αvβ6 cells were grown to approximately 30–40% confluence and transfected with mimics to miR-17-5p, miR-1281 (Thermo Scientific, Waltham, MA), a non-sense negative control miR (miR-NC), a control miR-342-5p that does not impact FMDV infection, or left untransfected following the RNAiMAX (Thermo Scientific, Waltham, MA) transfection method (manufacturer’s protocol). Forty-eight hours post-transfection, the transfected cells were inoculated with FMDV A24 Cruzeiro at a multiplicity of infection (MOI) of 10^−3^ and allowed to incubate for 24 h. The resulting virus titers were then determined for the collected samples following a previously described protocol [39]. Briefly, each sample was diluted 10^−1^ to 10^−6^ in virus growth medium (1% FBS, 1% A/A, and 25 mM HEPES in DMEM) and the dilutions were applied to LFBK-αvβ6 cell monolayers, incubated for 1 h at 37 ° C, acid-washed and rinsed, following by application of gum tragacanth overlay. The plates were then incubated overnight at 37 ° C, after which they were fixed and stained with crystal violent in Histochoice fixative (Amersco, Solon, OH). The plaques were counted and the calculated plaque forming units (PFU) per milliliter were plotted using Microsoft Excel (Microsoft Corporation, Redmond, WA).

## Results

### Differential regulation of miRNAs in serum during acute FMDV infection

To determine if FMDV infection of cattle (both acute and persistent) would induce an alteration in the abundance of circulating miRNAs in the serum, cattle were inoculated in triplicate with FMDV A24 Cruzeiro via the intra-nasopharyngeal (INP) route [40]. Serum was collected in acutely infected cattle at 3 dpi (viremic) as well as at 35 dpi in cattle that either cleared the virus (non-carriers) or became persistently infected with FMDV (carriers) (Fig. 1a). Serum was also collected from cattle prior to inoculation with FMDV as a baseline for comparison with infected animals. RNA was harvested and miRNA purified from the samples from which complementary DNA (cDNA) was generated via reverse transcriptase reaction (Fig. 1b). The resulting cDNAs were used as templates for RT-qPCR reactions on miRNA PCR array plates (Qiagen miScript Bovine miRNome) containing a series of primers unique to 169 characterized bovine miRNAs (Table 1) so as to identify miRNAs showing either up-regulation or down-regulation in the collected serum samples (Fig. 1c). The triplicate samples from each experimental set (acute, non-carrier, and carrier) were compared against uninfected controls, and first evaluated for quality of RT reaction and PCR efficiency; for which passing samples were then normalized against internal standards. Post-normalization, fold changes in miRNA levels were only accepted that exhibited statistical significance with *p*-values <0.05 across triplicate samples, and were only reported if a greater than 1.50-fold change was detected.

**Fig. 2:**
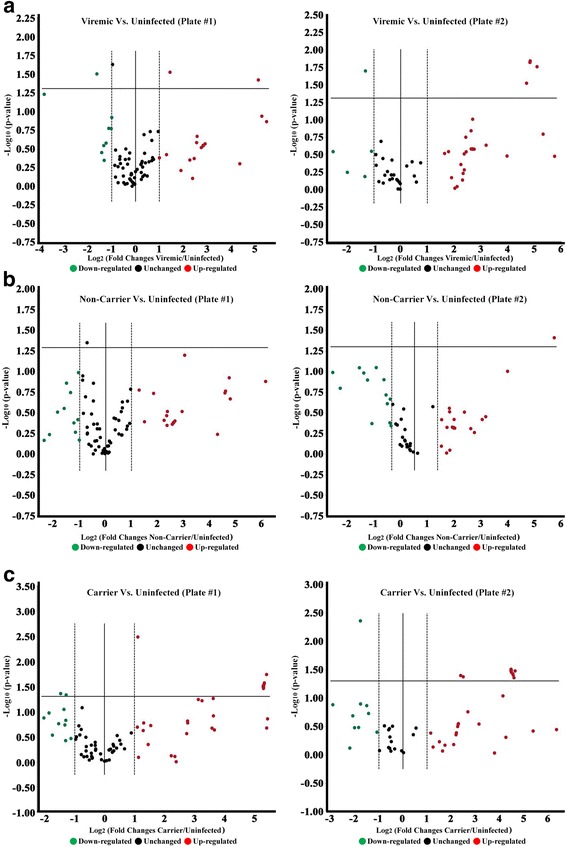
Differentially expressed miRNAs in response to FMDV infection. The expression patterns of 169 distinct bovine miRNAs was evaluated in serum harvested during three distinct phases of FMDV infection: **a** acute (viremic) **b** convalescent “non-carrier” and **c** persistently infected “carrier”. Expression levels were compared against serum from uninfected controls and are plotted onto volcano plots with the fold change in expression on the X-axis and the degree of reproducibility between replicates (p-value) on the Y-axis. Circles: miRNAs with unchanged expression, down-regulated expression (*green*), and up-regulated expression (*red*). miRNAs with significantly regulated expression (fold-change >1.5 and *p*-value of <0.05 [or greater than 1.25 by the –log_10]_) are plotted above the horizontal lines indicated on the volcano plots

Importantly, to avoid artifacts of technique, there was no pre-amplification of miRNAs prior to evaluation on the miRNA PCR arrays, which can potentially artificially increase levels of background miRNAs. By this approach, 7 bovine miRNAs were found up-regulated in serum collected from cattle acutely infected with FMDV (3–4 dpi) relative to uninfected controls. Raw scores are shown in Table 2, while volcano plots of log 2 fold changes in miRNA levels are shown in Fig. 2a. The up-regulated miRNA species included bta-miR-17-5p, bta-miR-146a, bta-miR-144, bta-miR-34a, bta-miR-369-3p, bta-miR-497, and bta-miR-22-5p (Table 2 and Fig. 2a). The three most up-regulated miRNAs were bta-miR-17-5p (+35.88 fold increase), bta-miR-146a (+34.36 fold increase), and bta-miR-144 (+28.78 fold increase) (Table 2). In contrast, three of the measured targets were significantly down-regulated during acute FMDV infection: bta-let-7 g (−1.96 fold decrease), bta-miR-1281 (−2.50 fold decrease), and bta-miR-26b (-3.09 fold decrease) (Table 2 and Fig. 2a). Some of the reported functionalities of these miRNA species are detailed in Table 2 to extrapolate for possible roles in acute FMDV infection.

**Table 2:** Bovine miRNAs up-regulated and down-regulated in cow serum in response to acute FMDV infection

miRNA regulation in Bovine serum during acute FMDV infection				
miRNA	Fold change	*p*-value	Ascribed function	Ref
miR-26b	−3.09	0.032	□ Role in adipogenesis and adipocyte differentiation □ Inhibits HBV replication by targeting CHORDC1 □ Inhibits HCV replication in PBMCs □ Promotes RSV replication by targeting TLR4 □ Tumor suppressor: targets USP9X, PTGS2, CDK8, TAK1, and TAB3	□ [69–76]
miR-1281	−2.50	0.020	□ Lipid metabolism: in dairy cattle, role in adipogenesis	□ [77]
let-7 g	−1.96	0.024	□ Cellular proliferation: targets lectin-like oxidized low density lipoprotein receptor-1 (LOX), caspase-3, and Aurora-B	□ [78–80]
miR-22-5p	+2.73	0.030	□ Tumor suppressor: targets CDK6, SIRT1, and Sp1	□ [81–88]
miR-497	+26.16	0.031	□ Tumor suppressor: targets CCNE1, insulin-like growth factor 1 receptor (IGF-1R), checkpoint kinase 1 (CHK1), represses eIF4E, E3 ubiquitin ligase	□ [89–93]
miR-369-3p	+28.57	0.015	□ Cellular proliferation: targets N-cadherin □ Down-regulates adipogenic differentiation in mesenchymal stromal cells	□ [94, 95]
miR-34a	+28.57	0.015	□ Tumor suppressor: targets CCND1, CDK6, FMNL2 and E2F5; activated by p53 □ Regulates lipid metabolism with HNF4α □ Immune modulatory: targets IL-6 and IL-8 □ Found in bovine milk	□ [96–101]
miR-144	+28.78	0.015	□ Tumor suppressor: targets CCNE1, ROCK1, ROCK2, EZH2, TIGAR, AKT3, E2F3, ADAMTS5, ADAM10, RAB14, and c-Met	□ [102–112]
miR-146a	+34.36	0.018	□ Tumor suppressor: targets SOS1 □ Up-regulated by HBV X protein □ Immune modulatory: induced by NF-KB signaling; targets IRAK1 and TRAF6, thus diminishing pro-inflammatory response from TLR signaling	□ [113–120]
miR-17-5p	+35.88	0.038	□ Cellular proliferation: targets SMAD7, FBXO31, E2F1, and c-Myc □ Repressed HCV infection, inversely correlated with HCV treatment response □ HIV-1 Tat C modulates NOX2 and NOX4 via miR-17 □ Immune modulatory: suppresses TLR signaling through IL6 and represses IFN-stimulated MxA expression	□ [121, 122]

List of bovine miRNAs that were observed to be up-regulated and down-regulated in serum samples collected during the acute phase of FMDV infection. Shown are the miRNA species, the fold-change in expression levels (in ascending order), and the ascribed function from literature searches of cattle miRNAs and homologous miRNAs from other species with little to no sequence divergence. The miRNAs listed showed a fold-change in expression levels of greater than 1.50, and had *p*-values of <0.05

### Differential regulation of miRNAs in serum during persistent FMDV infection

The FMDV carrier state is defined by continuous shedding of low quantities of infectious virus in oropharyngeal fluids beyond 28 dpi [41, 42]. This persistent phase of FMDV infection occurs in approximately 50–100% of FMDV-infected cattle, and is characterized by subclinical presence of actively replicating FMDV within the nasopharyngeal mucosa [34, 43, 44]. In contrast to acute FMDV infection, the persistent phase of infection is not associated with a marked local inflammatory reaction or a detectable systemic anti-viral response [34, 45]. Nine of the measured miRNAs were significantly up-regulated in persistently infected FMDV carriers compared to uninfected controls; and once again, raw scores are shown in Table 3, while volcano plots of log 2 fold changes in miRNA levels are shown in Fig. 2c. As shown in the top portion of Table 3: bta-miR-22-5p, bta-miR-147, bta-miR-1224, bta-miR-144, bta-miR-497, bta-miR-154a, bta-miR-17-5p, bta-miR-205, and bta-miR-31, with fold changes of 2.17, 5.28, 5.69, 23.78, 24.62, 24.05, 40.84, 41.22, and 43.37, respectively. The corresponding volcano plots for the two array plates are shown in Fig. 2c. Bta-miR-205 was the strongest induced miRNA during persistent infection. Three miRNAs were significantly down-regulated in serum from persistently infected cattle: bta-miR-1281, bta-miR-181b, and bta-miR-23b-5p with fold decreases of 3.41, 2.77, and 2.44, respectively (Table 3). The strongest down-regulated miRNA, miR-1281, was also observed to have decreased levels in the serum collected from acutely infected cattle.

**Table 3:** Bovine miRNAs up-regulated and down-regulated in cow serum in response to persistent FMDV infection

miRNA regulation in Bovine serum during persistent FMDV infection				
Persistently Infected FMDV Carriers				
miRNA	Fold change	*p*-value	Ascribed function	Ref
miR-1281	−3.41	0.004	□ Lipid metabolism: in dairy cattle, role in adipogenesis	□ [50]
miR-181b	−2.77	0.044	□ Cellular proliferation: targets RASSF1A and NF-KB □ Immune modulatory: adenylyl cyclase 9 (AC9), antagonistic to IFNα expression	□ [123–126]
miR-23b-5p	−2.44	0.047	□ Tumor suppressor: up-regulated by p53, targets mitochondrial glutaminase, antagonizes c-Myc signaling, and suppresses metastasis □ Cellular proliferation: targets proline oxidase	□ [127–132]
miR-22-5p	+2.17	0.003	□ Tumor suppressor: targets CDK6, SIRT1, and Sp1 □ Circulating miRNA bio-marker for Huntington’s Disease and acute phase myocardial infarction □ Involved in neonatal heart development □ Anti-inflammatory: negatively regulates type I IFN inflammatory cytokine response	□ [82–86, 88]
miR-147	+5.28	0.039	□ Anti-inflammatory: TLR2, TLR3, and TLR4 stimulate miR-147 expression, which participates in negative feedback loop to suppress inflammatory cytokine expression □ Up-regulated by HCV Genotype-4 □ Tumor suppressor: targets HOXC6 oncogene	□ [133–135]
miR-1224	+5.69	0.042	□ Tumor suppressor: targets CREB1	□ [136]
miR-144	+23.78	0.037	□ Tumor suppressor: targets cyclin E1 (CCNE1), ROCK1, ROCK2, SMAD4, TIGAR, E2F3, ADAMTS5, ADAM10, RAB14, ZEB1/2, and c-Met	□ [103–112]
miR-154a	+24.05	0.039	□ Tumor suppressor: targets E2F5, ZEB2, Wnt5a, Wnt11, TLR2, and HMGA2	□ [137–143]
miR-497	+24.62	0.044	□ Tumor suppressor: targets CCNE1, insulin-like growth factor 1 receptor (IGF-1R), checkpoint kinase 1 (CHK1), represses eIF4E, E3 ubiquitin ligase	□ [89–92, 144]
miR-17-5p	+40.84	0.029	□ Cellular proliferation: targets SMAD7, FBXO31, E2F1, and c-Myc □ Repressed HCV production, inversely correlated with HCV treatment response □ HIV-1 Tat C modulates NOX2 and NOX4 via miR-17-5p □ Immune modulatory: suppresses TLR signaling through IL-6 and represses IFN-stimulated MxA expression	□ [121, 122]
miR-205	+41.22	0.027	□ De-regulates lipid metabolism by targeting ACSL1 □ Cellular proliferation: targets ZEB1, ZEB2, Ubc13, PTEN, IL13RA2, COL5A2, ADM, CXCR2, XPO6, SPSB1, FMO5, and PSMF1 □ HBV X protein hyper-methylates promoter of miR-205 to suppress apoptosis	□ [145, 146]
miR-31	+43.37	0.018	□ Cellular proliferation: up-regulated in tumor cells □ Downstream target genes of miR-31 have an effect on lipid metabolism and adipogenesis □ Innate immunity related: targets MyD88 and interferes with TLR2 and TLR4 signaling	□ [147–151]
Convalescent Cattle (Non-Carriers)				
miR-455-3p	+68.17	0.039	□ Tumor suppressor: targets RAF	□ [61]
miR-150	−1.65	0.044	□ Immune modulatory: targets MyD88 (key TLR regulator) and CXCR4	□ [147]

List of bovine miRNAs that were observed to be up-regulated and down-regulated in serum samples collected from cattle persistently infected with FMDV (“FMDV carriers”) and convalescent cattle that had successfully cleared infection (“non-carriers”). Differential miRNA expression is presented relative to uninfected animals. Shown are the miRNA species, the fold-change in expression levels (in ascending order), and the ascribed function from literature searches of cattle miRNAs and homologous miRNAs from other species with little to no sequence divergence. The miRNAs listed showed a fold-change in expression levels of greater than 1.50, and had *p*-values of <0.05

As could be expected, the miRNA expression profile in cattle that had successfully cleared FMDV infection was more similar to that of the uninfected controls. In this cohort of animals, only two miRNAs were differentially expressed compared to the baseline: bta-miR-150 and bta-miR-455-3p, which were down-regulated 1.65 and up-regulated 68.17 fold, respectively (Table 3). Notably, bta-miR-455-3p was the highest up-regulated miRNA detected in the entire proof-of-concept study; see outlying miRNA in the far right of the volcano plot for array plate 2 (Fig. 2b). The non-carrier results reinforce the viability of this miRNA profiling approach described in this proof-of-concept study given that virtually all of the miRNA species have returned to baseline levels. As mentioned in the previous section, some of the reported functionalities associated with these miRNAs is described in Table 3, allowing for hypothetical contributions to be formulated.

### Genomic localization of the differentially expressed miRNAs

**Fig. 3:**
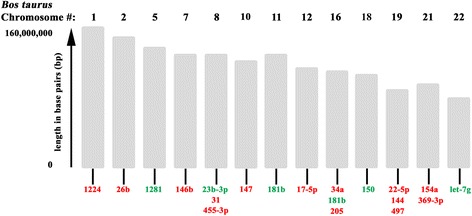
Genomic localization of miRNAs differentially expressed in response to FMDV infection. The genomic localization of the bovine miRNAs that were found to be differentially expressed in this study were examined on miRbase (current version) for which chromosomes they localized to in the *Bos taurus* genome. Bovine chromosomes on which miRNA sequences localized were arrayed numerically with the chromosome size in base pairs displayed. miRNAs were listed by their numerical designation in red if down-regulated and in green if up-regulated

To further characterize the miRNAs that were found to be differentially regulated in response to FMDV infection, we sought to determine if there was any correlation between the miRNAs with respect to where their sequences map in the *Bos taurus* genome. To that end, the miRNAs were queried in the most current version of miRbase (release 21, June 2014, miRBase.org) for their chromosomal sequence sites. As shown in Fig. 3, the encoded sequences of the miRNAs detected in this study were found scattered across bovine chromosomes: 1, 2, 5, 7, 8, 10, 11, 12, 16, 18, 19, 21, and 22. The only chromosomes in the *Bos taurus* genome that were associated with more than one of the identified miRNAs were: chromosome #8 with bta-miR-23b-5p, bta-miR-31, and bta-miR-455-3p; chromosome #16 with bta-miR-34a, bta-miR-181b, and bta-miR-205; chromosome #19 with bta-miR-22-5p, bta-miR-144, and bta-miR-497; and finally chromosome #21 with bta-miR-154a and bta-miR-369-3p. The miRBase database was also queried for whether the gene sequences for the identified miRNAs were localized to intergenic or intronic regions of the *Bos taurus* genome. Eleven of the miRNAs are encoded in intergenic regions, including: bta-miR-1281, bta-miR-150, bta-miR-181b, bta-miR-497, bta-miR-144, bta-miR-34a, bta-miR-154a, bta-miR-146b, bta-miR-17-5p, bta-miR-205, and bta-miR-31. The remaining 8 miRNAs are encoded within intronic regions: bta-miR-26b, bta-miR-455-3p, bta-miR-23b-5p, bta-let-7 g, bta-miR-22-5p, bta-miR-147, bta-miR-369-3p, and bta-miR-1224. Based on these findings, we concluded that there was no pattern to where in the bovine genome the detected miRNAs were expressed.

### Cluster analysis of the differentially expressed miRNAs

**Fig. 4:**
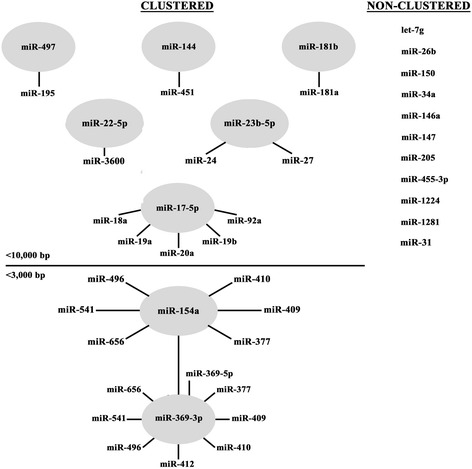
Cluster analysis of miRNAs differentially expressed in response to FMDV infection. Two different databases were employed to assess whether the differentially expressed miRNAs detected in this study were clustered or non-clustered: miRbase (current version) and MetaMirClust. The miRNAs found to be non-clustered were listed to the right. The miRNAs determined to be clustered were shown in circles with clustered miRNA species indicated around the circle. With the exception of bta-miR-369-3p with a cutoff of <3000 bp, the distance cutoff between miRNA sequences for the cluster analysis was set at <10,000 bp

Many miRNA sequences encoded in the genomes of various species have been discovered clustered with other miRNAs that often share similar regulatory functions [46, 47]. Given that, cluster analysis was performed on the differentially regulated bovine miRNAs detected in this study using two different sources: miRbase (release 21, June 2014, miRBase.org) and MetaMirClust [48]. These two databases provided corroborating data regarding whether the detected miRNAs were non-clustered or clustered, and what miRNAs clustered with them. As shown in Fig. 4, 11 of the miRNAs that were observed to be differentially regulated in the serum from cattle during FMDV infection were not clustered with other miRNAs in the *Bos taurus* genome. The non-clustered miRNAs included: let-7 g, bta-miR-26b, bta-miR-150, bta-miR-34a, bta-miR-146a, bta-miR-147, bta-miR-205, bta-miR-455-3p, bta-miR-1224, bta-miR-1281, and bta-miR-31. The remaining 8 miRNAs (bta-miR-497, bta-miR-144, bta-miR-181b, bta-miR-22-5p, bta-miR-23b-5p, bta-miR-17-5p, bta-miR-154a, and bta-miR-369-3p) detected in this study were found to be clustered. Of these 8, bta-miR-154a and bta-miR-369-3p were the most heavily clustered miRNAs, which is why a more stringent cluster distance was imposed of <3,000 bp apart. These two miRNAs were also the only two from this study that were clustered with each other. Similar to the genomic localization of the detected miRNAs, we concluded that there was no pattern in the clustering data.

### Effect of miR-17-5p and miR-1281 on the progression of FMDV infection in vitro

**Fig. 5:**
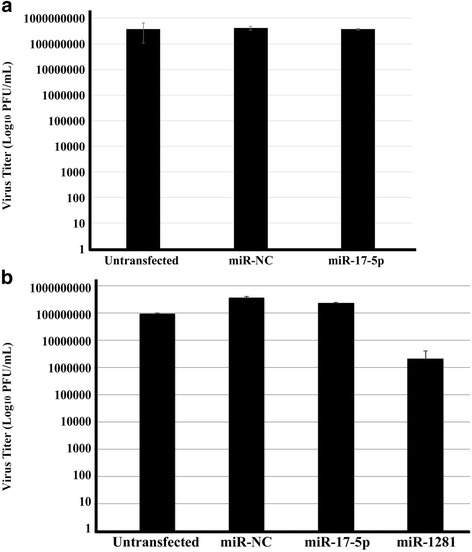
Effect of miR-17-5p and miR-1281 mimics on FMDV infection in cell culture. **a** Cells were transfected with miR-17-5p mimics in parallel with negative controls: untransfected and irrelevant miR-342-5p. Forty-eight hours post-transfection, the cells were infected with FMDV A24 Cruzeiro. Subsequent to infection, the resulting samples were evaluated for virus titers, which are plotted. **b** Cells were transfected with miR-1281 mimics following the same procedure as miR-17-5p (**a**). Resulting virus titers are plotted as shown. Both panels **a** and **b** are representative of two independent experiments that were performed in duplicate. miR-17-5p was included in the second experimental set in panel **b** to confirm the non-effect seen in panel **a**

At the completion of the miRNA profiling analysis, we sought to evaluate the individual impact of one of the up-regulated and one of the down-regulated miRNA species identified on FMDV infection in a cell culture system. Of the miRNA species detected, miR-17-5p and miR-1281 were selected as they were among the 5 miRNA that showed similar differential expression in two different animal sets: acutely infected cattle (Table 2 and Fig. 2) and persistently infected cattle (Table 3 and Fig. 2). To that end, mimics of miR-17-5p were transfected into LFBK-αvβ6 cells, a cell line commonly used for FMDV propagation [37, 38]. Two days post-transfection, cells were infected with FMDV strain A24 Cruzeiro (the same FMDV strain that was used to infect cattle in the current study). It was hypothesized that the elevated expression observed for miR-17-5p in serum samples collected from acutely as well as persistently infected cattle could indicate that this miRNA was either induced by FMDV infection to benefit virus replication, or, induced as a consequence of the host response aiming to suppress virus infection. In the in vitro experiment, there was no impairment to virus replication in cells transfected with miR-17-5p prior to FMDV infection, and virus titers produced were nearly identical to that of the negative controls (untransfected cells and cells transfected non-sense miRNA mimics [miR-NC]) used in the experiment (Fig. 5a).

In a separate experiment, we investigated the potential impact of one of the down-regulated miRNAs that was common to both acutely and persistently infected cattle, miR-1281, on the progression of FMDV infection in vitro. As described for miR-17-5p above, mimic molecules of miR-1281 were transfected into LFBK-αvβ6 cells and 2 days post-transfection, the cells were infected with FMDV A24 Cruzeiro. In contrast to the findings with miR-17-5p, the transfection of miR-1281 prior to FMDV infection had a negative impact on the progression of FMDV infection confirmed by a 2 to 3 log reduction in viral titers observed in transfected cells compared to controls (Fig. 5b). These in vitro findings are consistent with the results of the in vivo miRNA-profiling study, in that an invading virus (in this case FMDV) would likely attempt to induce the down-regulation of miRNAs that would be detrimental to its replication cycle, while up-regulating those that would be beneficial.

## Discussion

**Fig. 6:**
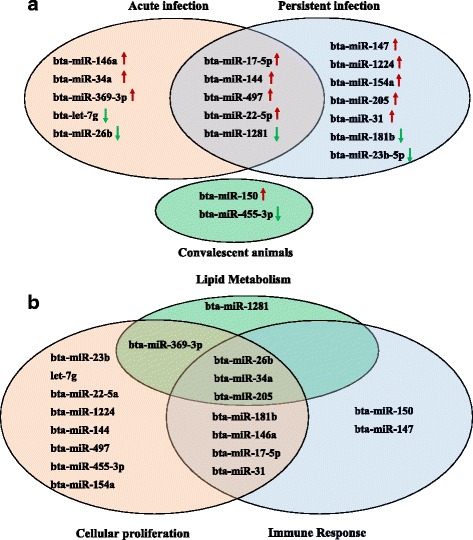
Schematic overview of the general function of the miRNAs found to be differentially expressed in cattle serum in response to FMDV infection. **a** The miRNAs identified in the study that are unique to acute infection (*orange circle*), persistent infection (*blue circle*), and convalescent animals (*green circle*) are shown, as well as those shared between acutely and persistently infected cattle. *Red and green arrows* indicate if targets were up- or downregulated. **b**. The functionality of the miRNAs detected could be separated into three general categories: cellular proliferation (*orange circle*), immune response (*blue circle*), and lipid metabolism/adipogenesis (*green circle*). A Venn diagram was constructed showing which miRNAs exhibited functionality exclusive to one category and which miRNAs overlapped into more than one of the aforementioned categories

In viral disease research, miRNA profiling has become a topic of considerable interest as researchers search for cellular targets that can be exploited in order to combat infection. In the case of FMDV, the sole miRNA profiling study to date was an investigation of the differential expression of miRNAs in a porcine based cell line in response to infection with an isolate of FMDV serotype Asia1 [49]. The widespread utility of these findings is somewhat limited given that cell culture is not necessarily reflective of the molecular pathogenesis of the virus in an in vivo setting. miRNA profiling analysis of tissue, urine, and serum samples from infected organisms is being conducted with greater frequency. Using miRNA RT-qPCR profiling arrays containing 169 characterized miRNAs found in *Bos taurus*, we investigated the differential levels of miRNAs in sera collected from cattle during three distinct phases of infection with FMDV serotype A isolate A24 Cruzeiro, including acute and persistent phases of infection. Moreover, persistent phase samples were obtained from sub-clinically infected FMDV carriers as well as convalescent animals that had successfully cleared the infection. A total of 19 miRNAs exhibited an altered abundance in serum in response to FMDV infection. Five of the miRNAs identified with significant shifts in circulating levels relative to uninfected animals were unique to acutely infected cattle (Fig. 6a, orange circle), seven were only observed in serum from persistently infected carriers (Fig. 6a, blue circle), whereas two were specific to convalescent animals (Fig. 6a, green circle). Of the miRNAs that were significantly up- or down-regulated, five were shared between acutely and persistently infected cattle (bta-miR-17-5p, bta-miR-144, bta-miR-497, bta-miR-22-5p, and bta-miR-1281). Mimics of miR-17-5p and miR-1281 were separately tested for their effect on FMDV replication in cell culture (Fig. 5). While miR-17-5p had no apparent impact on the progression of FMDV infection, mimics of miR-1281 decreased the resulting FMDV titers by 2–3 logs. From these findings, it could be inferred that the induction of miR-17-5p in vivo might manipulate the cellular environment such that it favors FMDV replication, while miR-1281 is down-regulated due to an antagonistic effect. Given that miR-1281 mimics impede FMDV replication and the reported role of miR-1281 in cattle [50], it seems that the state of lipid homeostasis in the host plays an important role in the outcome of FMDV infection regardless of whether it is acute or persistent. A survey of all the predicted target genes of these two miRNAs using multiple online algorithms failed to produce a consensus target to be explored in this current study (see Additional file 2: Table S1).

The previously reported miRNA profile of a porcine cell line infected with FMDV serotype Asia1 was obtained using high throughput sequencing [49], which represents the first time such a differential profiling experiment was performed for FMDV. The present study represents the first miRNA profiling experiment, albeit a limited small scale proof-of-concept study, using serum collected from uninfected and infected cattle. This study also marks the first time this type of analysis was performed for a serotype A FMDV isolate (A24 Cruzeiro) as well as the first time differentially expressed miRNAs were examined in the context of persistent FMDV infection. Four of the miRNAs detected were shared between the two profiling studies: miR-22-5p, miR-146b, miR-23b-5p, and miR-369-3p. Notably, these four miRNAs all participate in the regulation of cell proliferation and apoptosis. These may also potentially represent four miRNAs that are distinctive to FMDV infection across all serotypes, while the targets not shared between these two studies represent sets of miRNAs that are either specific to the FMDV strain or the experimental system (cell culture versus infected cattle). Future profiling studies with other FMDV serotypes will be necessary to bring certainty to these suppositions. We also wanted to examine the differential miRNA profiles in serum from cattle infected with other viral pathogens; however, we were unable to find published reports comparable to the proof-of-concept study described herein. Despite that, the miRNA profiles generated from human serum collected from individuals infected with related + ssRNA viruses (Dengue virus and HCV) showed some dysregulated miRNAs shared with the ones reported for FMDV here, which included: let-7 g, miR-22-5p, miR-23b-5p, miR-146a, and miR-497 [51, 52]. Of note, these shared miRNAs all are reportedly involved in the signaling pathways involved in cellular proliferation and apoptosis, from which it can be inferred that these pathways are important to life cycle of + ssRNA viruses. Potentially, these shared miRNAs that are differentially regulated in the serum in response to invasion by + ssRNA viruses suggest that common molecular mechanisms are activated or suppressed to either combat + ssRNA viruses or to alter the host environment such to facilitate + ssRNA virus replication.

There is increased evidence to support the value of the application of profiling of miRNAs detectable in circulation as biomarkers suggesting the presence and even prognosis of specific disease conditions. For example, hand-foot-and-mouth disease (HFMD) results from infection by one of many possible Picornaviruses, and research has demonstrated that each of these viruses elicits a unique systemic miRNA response that enables differentiation of the potential causative agents [17]. Thus, it would be of interest to elucidate the signature miRNA response for FMDV infection, which may be applicable across all serotypes of FMDV or could be different with each of the seven known serotypes and in different host organisms such as goats, sheep, and pigs. Similarly, identification of distinct changes in circulating miRNAs potentially associated with subclinical persistence of FMDV could be of great value for development of novel diagnostic approaches for disease surveillance. Furthermore, some of the miRNAs detected in this study might also serendipitously target regions of the FMDV genome, and could be potentially adapted as bio-therapeutic against FMDV similar to recent report with miR-203a-3p and miR-203a-5p [53]. To briefly explore that possibility, a bioinformatics analysis was conducted using two miRNA recognition sequence mapping algorithms (miRmap and ViTa) [54, 55], where the sequence for the FMDV isolate used in this study was input (A24 Cruzeiro, Accession #AY593768) along with each individual miRNA identified herein (see Additional file 3: Table S2). The miRmap algorithm reported that 6 of the 19 miRNAs dysregulated in bovine serum in response to FMDV could potentially target different regions of the FMDV A24 Cruzeiro RNA genome: bta-miR-17-5p, bta-miR-497, bta-miR-146a, bta-miR-1224, bta-miR-31, and bta-miR-150. In contrast, the ViTa algorithm found that more of these miRNAs could potentially target the genome, adding bta-miR-205, bta-miR-26b, bta-let-7 g, bta-miR-34a, bta-miR-144, bta-miR-181b, and bta-miR-147 to the list. Future studies with these new miRNA targets will certainly need to explore the relative stability of the FMDV genome in the presence of elevated levels of these select miRNAs.

While approximately 87% of the profiled miRNAs exhibited no change in circulation levels between uninfected and infected animals, roughly 13% were significantly up-regulated or down-regulated as a result of FMDV infection. Of the differentially regulated miRNAs, 16 (bta-miR-23b-5p, let-7 g, bta-miR-22-5p, bta-miR-1224, bta-miR-144, bta-miR-497, bta-miR-455-3p, bta-miR-154a, bta-miR-369-3p, bta-miR-26b, bta-miR-34a, bta-miR-205, bta-miR-181b, bta-miR-146a, bta-miR-17-5p, and bta-miR-31) have previously been described to play a role in cellular proliferation or apoptosis (Fig. 6b, orange circle). Nine of the miRNAs (bta-miR-26b, bta-miR-34a, bta-miR-205, bta-miR-181b, bta-miR-146a, bta-miR-17-5p, bta-miR-31, bta-miR-150, and bta-miR-147), have been ascribed immune modulatory functions (Fig. 6b, blue circle). Also notable is that five of the detected miRNAs (bta-miR-1281, bta-miR-369-3p, bta-miR-26b, bta-miR-34a, and bta-miR-205) are involved in adipogenesis or other lipid metabolic pathways (Fig. 6b, green circle). Interestingly, there were an equivalent number of immune modulatory miRNAs differentially expressed between acutely and persistently infected cattle with one down-regulated and three up-regulated. Only one of the immune modulatory miRNAs was shared between the two sets: bta-miR-17-5p, which was significantly upregulated during both acute and persistent FMDV infection. This miRNA has been extensively studied for its roles in immune modulation as well as cellular proliferation and apoptosis [56]. Additionally, miR-17-5p has been implicated in T-cell activation, B-cell and monocyte maturation as well as with suppression of TLR signaling and hampering of the IFN response [16, 57, 58], which are important functions of the host anti-viral response. These two test groups also exhibited approximately the same number of miRNAs associated with cell proliferation and apoptosis with eight and ten differentially expressed in acutely and persistently infected animals, respectively. Interestingly, there were two times more lipid metabolism-related miRNAs with dysregulated expression in acutely infected cattle, suggesting that modulation of such pathways is important to active FMDV replication. It is known that the VP4 structural protein is acylated with myristate [59]; and more recently, a SILAC screening showed that LYPLA, which is a known depalmitoylase, is a critical factor in FMDV infection [60]. It would be expected that the serum miRNAs of an animal that cleared FMDV would return to a basal level similar to an uninfected animal, and consistent with that, the samples obtained from convalescent cattle exhibited only two dysregulated miRNAs: one that is immune modulatory (bta-miR-150) and one that promotes cellular proliferation (bta-miR-455-3p). In serum from convalescent animals, bta-miR-150 was marginally down-regulated, possibly indicating a reduction in immune system activation given that the FMDV infection was cleared. In strong contrast, bta-miR-455-3p was the most up-regulated miRNA in all of the data sets examined in this study. A survey of published findings on miR-455-3p point toward it being a potent upstream regulator of multiple signaling pathways associated with induced cellular proliferation [61–63]. Since one of the cellular mechanism for combating a viral infection is the induction of apoptosis in infected cells, thereby also increasing cellular turn-over, the augmentation in bta-miR-455-3p expression may be reflective of the animal reversing those effects and shutting down those pathways now that FMDV infection has been eliminated. As with the bioinformatics analysis conducted to identify prospective gene targets for miR-17-5p and miR-1281, a consensus target gene for miR-455-3p was not found to further investigate (Additional file 2: Table S1). However, consistent with the speculation above, many of the target genes proposed for miR-455-3p were cell cycle regulators and zinc-finger containing proteins, from which it can be inferred that this single miRNA was regulating multiple cell survival pathways to facilitate the final stages of clearing FMDV infection.

It is interesting to note that six of the 19 miRNAs described in this study are considerably abundant in cattle liver: bta-miR-22-5p, bta-miR-150, bta-miR-17-5p, bta-miR-455-3p, bta-miR-146, and let 7-g [64]; an organ in which FMDV does not establish infection. However, due to the substantial blood flow through the liver, large quantities of FMDV particles will pass through the liver during high titer viremia. It is unclear if or how FMDV may be affecting this tissue to possibly stimulate the secretion of the aforementioned miRNAs into the circulation. However, acute FMDV infection in cattle has previously been associated with a strong acute phase response characterized by significant induction of serum amyloid A and haptoglobin levels in serum [65]. These proteins are secreted from the liver in response to a systemic inflammatory reaction [66], which further suggests that the systemic effect of FMDV infection may affect hepatic gene expression and protein synthesis. Further investigation will be required to elucidate the extent and potential application of these findings.

In conclusion, we have identified several miRNAs that are elevated or diminished in circulation of cattle that have been infected with FMDV. Interestingly, unique miRNA serum signatures were detected for acutely infected and persistently infected cattle. The unique miRNAs found in serum from persistently infected cattle may help determine what cellular pathways are actively contributing to the development and maintenance of the “FMDV carrier state”. Characterization of signature serum miRNAs that are distinct to the FMDV carrier state could facilitate identification of persistently infected animals in post-outbreak surveillance. Moreover, the miRNAs described may also be adapted as potential anti-viral therapeutics as has been attempted with siRNAs in the past [6–9, 67, 68]. Further investigations including increased sample numbers will be needed to verify the findings of the current proof-of-concept study. Additional in vitro investigations may provide further knowledge of distinct biological functions and potential therapeutic applications of the bovine miRNAs identified herein.

## Conclusions

Perturbations in the circulating levels of miRNAs in the bloodstream of humans and animals in response to infection can potentially provide diagnostic and prognostic value. Here, for the first time in a small scale proof-of-concept study, differentially regulated serum miRNAs from cattle infected with FMDV at different phases of infection were reported. Signature circulating miRNAs were identified for acutely and persistently infected animals, which could be applied to the development of diagnostic assays allowing for distinction between carrier and non-carrier cattle.

## Additional files

Additional file 1: Table S3. Cattle serum samples used for the miRNA profiling study. (DOCX 12 kb)

Additional file 2: Table S1. Predicted mRNA targets for indicated miRNAs. (DOCX 21 kb)

Additional file 3: Table S2. Predicted FMDV genomic targets for indicated miRNAs. (DOCX 15 kb)

## Abbreviations

FMDV: Foot-and-mouth disease virus
miRNA, miR: microRNA
