# Supplementary material for: Proof-of-concept study: profile of circulating microRNAs in Bovine serum harvested during acute and persistent FMDV infection
Source: Virol J. 2017 Apr 7;14:71. doi: 10.1186/s12985-017-0743-3 (PMC5384155; doi:10.1186/s12985-017-0743-3)
Supplement: Supplementary file 1 — Cattle serum samples used for the miRNA profiling study. (DOCX 12 kb) [file 12985_2017_743_MOESM1_ESM.docx]

**Additional file 1: Table S3. Cattle serum samples used for the miRNA profiling study.**

| **Animal ID #** | **Sample Type** | **dpi** | **Description** |
| --- | --- | --- | --- |
| BR14-25 | Serum | 0 | Negative Serum  (collected pre-inoculation) |
| BR14-32 | Serum | 0 | Negative Serum  (collected pre-inoculation) |
| BR14-106 | Serum | 0 | Negative Serum  (collected pre-inoculation) |
| BR14-22 | Serum | 2 | Viremic Serum |
| BR14-25 | Serum | 3 | Viremic Serum |
| BR14-44 | Serum | 4 | Viremic Serum |
| BR14-106 | Serum | 21 | Carrier Serum |
| BR14-110 | Serum | 35 | Carrier Serum |
| BR14-30 | Serum | 35 | Carrier Serum |
| BR14-32 | Serum | 35 | Non-Carrier Serum |
| BR14-108 | Serum | 35 | Non-Carrier Serum |
| BR14-111 | Serum | 35 | Non-Carrier Serum |

*dpi: days post-inoculation
