# Supplementary material for: Proof-of-concept study: profile of circulating microRNAs in Bovine serum harvested during acute and persistent FMDV infection
Source: Virol J. 2017 Apr 7;14:71. doi: 10.1186/s12985-017-0743-3 (PMC5384155; doi:10.1186/s12985-017-0743-3)
Supplement: Supplementary file 2 — Predicted mRNA targets for indicated miRNAs. (DOCX 21 kb) [file 12985_2017_743_MOESM2_ESM.docx]

**Additional file 2: Table S1. Predicted mRNA targets for indicated miRNAs.** The two miRNAs (miR-17-5p and miR-1281) that were examined *in vitro* for their individual effect on FMDV replication were also investigated for their potential cellular target genes through a bioinformatics approach using 4 different online miRNA target prediction algorithms: DIANA-TarBase (1), miRDB (2), miRanda (3), and miRmap (4). The top 20 predicted target genes were compiled for each algorithm in the table below.

| **DIANA-TarBase v7.0**  **Vlachos et al., (2014) *Nucleic Acids Research*** | **miRDB.org**  **Wang et al., (2016) *Bioinformatics*** | **miRanda-microRNA.org**  **Betel et al., (2008) *Nucleic Acids Research*** | **miRmap**  **Vejar and Zdobnov, (2012) *Nucleic Acids Research*** |
| --- | --- | --- | --- |
|  |  |  |  |
| **miR-1281** | | | |
| **GENE TARGETS** | **GENE TARGETS** | **GENE TARGETS** | **GENE TARGETS** |
| ERO1L | GCLM | HCG9 | LHFPL4 |
| HN1L | FAM101A | NGDN | KLHL29 |
| EIF5 | ST3GAL2 | PTS | SLC35B2 |
| SOX11 | PDXP | C2orf39 | MCC |
| CCND1 | MFSD7 | SLC6A14 | GRK5 |
| ZNF552 | ERO1L | CXorf66 | SEC63 |
| EPG5 | PTPRD | C4orf51 | HIST1H1E |
| PPM1B | DLGAP4 | EED | PTPN1 |
| RAP1A | CXorf66 | GHRL | PGM2 |
| PVRL4 | C6orf106 | C9orf89 | PHC2 |
| EMP2 | RTN2 | LDB2 | ASAH1 |
| MYO18A | SYNCRIP | NRG1 | BOK |
| ATP8B1 | EPO | EPO | TSR1 |
| IVD | C22orf34 | ADCK2 | VSTM2L |
| NDUFV1 | DAG1 | SPANXN5 | SIL1 |
| MLLT6 | TLDC2 | C4orf29 | DNAJC18 |
| CLTC | SLC12A5 | SORBS2 | ANO10 |
| HNRNPF | CCDC142 | RND3 | USP46 |
| ARHGEF7 | S1PR3 | ERO1L | GBA2 |
| ALS2 | ZBTB46 | TMC2 | INOS |
|  |  |  |  |
| **miR-17-5p** | | | |
| **GENE TARGETS** | **GENE TARGETS** | **GENE TARGETS** | **GENE TARGETS** |
| MAP3K9 | ZNF800 | DYNC1LI2 | ANKRD17 |
| RGMB | ARID48 | PLEKHA3 | MGC157163 |
| EFCAB14 | ADARB1 | BRMS1L | FAM3C |
| ARAP2 | PTPN4 | ZNFX1 | DSG1 |
| ZNF148 | PKD2 | MYT1L | PKD2 |
| TBC1D9 | GAB1 | EPHA4 | USP46 |
| STAT3 | SLC40A1 | ENPP5 | TMEM50B |
| ANKRD13C | ZNFX1 | SLITRK3 | MAPK8 |
| ARNTL2 | FBXL5 | RPS6KA5 | FAM45A |
| SUV420H1 | EPHA4 | SERF1B | UBR5 |
| CCND2 | PDCD1LG2 | CAMTA1 | GNS |
| PRR15 | EPHA5 | C14orf28 | RRAGD |
| DCAF10 | FGD4 | ANUBL1 | PFN2 |
| MEX3D | EZH1 | RUFY2 | ANGEL2 |
| LSM14B | STK17B | SUV420H1 | BRMS1L |
| STRN4 | ARID4A | GNPDA2 | AHCTF1 |
| SYTL4 | GPR137C | SCN1A | TNFRSF21 |
| GRAMD1A | USP3 | SERF1A | VMA21 |
| SUSD1 | SALL1 | RPGR | SLC46A3 |
| ECT2 | MASTL | LHX8 | PLSCR4 |
|  | | | |
| **miR-455-3p** | | | |
| **GENE TARGETS** | **GENE TARGETS** | **GENE TARGETS** | **GENE TARGETS** |
| BRWD1 | STK17B | SS18 | TYK2 |
| H1F1AN | ARMC8 | TMEFF1 | CUL3 |
| ARMC8 | CUL3 | SUCLA2 | GNS |
| TP73 | PIK3R1 | RNF152 | ZNF266 |
| ASB1 | NAA30 | TPRG1 | TNFSF18 |
| C11orf58 | GABARAPL2 | LOC64301 | CD93 |
| PDS5B | NUP153 | TLE4 | C19H17orf108 |
| RASA2 | HOXC4 | GBAS | MOSPD1 |
| CENPA | PHF6 | TFAP2A | CTNND1 |
| S100PBP | UHRF1BP1 | AP1S2 | CPPED1 |
| CSNK1A1 | ZNF12 | PAFAH1B2 | ANKRD26 |
| SLC35G2 | TTK | DNAJB12 | ENSBTAT |
| DOK4 | PLA2G15 | ARMC8 | MRPL53 |
| ZNHIT6 | UBE2Q2 | TMEM155 | SSR1 |
| RUNDC1 | ELF3 | ZNF238 | PDHA1 |
| ARHGEF37 | SAR1A | ADCK1 | ATXN7L3B |
| CKAP2 | ZCCHC10 | PBRM1 | KIAA0226L |
| LONRF2 | RMND5A | NCRNA00230B | GCFC |
| RFWD3 | STX6 | ABCB1 | MAPK3 |
| IGFBP3 | KPNA3 | AUTS2 | CBLN2 |

1. **Vlachos IS, Paraskevopoulou MD, Karagkouni D, Georgakilas G, Vergoulis T, Kanellos I, Anastasopoulos IL, Maniou S, Karathanou K, Kalfakakou D, Fevgas A, Dalamagas T, Hatzigeorgiou AG.** 2015. DIANA-TarBase v7.0: indexing more than half a million experimentally supported miRNA:mRNA interactions. Nucleic Acids Res **43:**D153-159.

2. **Wang X.** 2016. Improving microRNA target prediction by modeling with unambiguously identified microRNA-target pairs from CLIP-ligation studies. Bioinformatics **32:**1316-1322.

3. **Betel D, Wilson M, Gabow A, Marks DS, Sander C.** 2008. The microRNA.org resource: targets and expression. Nucleic Acids Res **36:**D149-153.

4. **Vejnar CE, Zdobnov EM.** 2012. MiRmap: comprehensive prediction of microRNA target repression strength. Nucleic Acids Res **40:**11673-11683.
