# Supplementary material for: Proof-of-concept study: profile of circulating microRNAs in Bovine serum harvested during acute and persistent FMDV infection
Source: Virol J. 2017 Apr 7;14:71. doi: 10.1186/s12985-017-0743-3 (PMC5384155; doi:10.1186/s12985-017-0743-3)
Supplement: Supplementary file 3 — Predicted FMDV genomic targets for indicated miRNAs. (DOCX 15 kb) [file 12985_2017_743_MOESM3_ESM.docx]

**Additional file 3: Table S2. Predicted FMDV genomic targets for indicated miRNAs.** The RNA sequence of the genome of FMDV isolate A24 Cruzeiro (Accession #AY593768) was analyzed for possible recognition sequences in the genome for the miRNAs detected in this proof-of-concept profiling study using the miRmap (1) and ViTa (2) online algorithms.

| **microRNA** | **miRmap** | **Target Site** | **ViTa** | **Target Site** |
| --- | --- | --- | --- | --- |
| miR-17-5p | YES | 5482 | YES | 5482 |
| miR-1281 | NO |  | NO |  |
| miR-455-3p | NO |  | NO |  |
| miR-205 | NO |  | YES | 5929, 3729 |
| miR-26b | NO |  | YES | No target given |
| let-7g | NO |  | YES | No target given |
| miR-22-5p | NO |  | NO |  |
| miR-497 | YES | 6875, 5963, 726 | YES | 6875 |
| miR-369-3p | NO |  | NO |  |
| miR-34a | NO |  | YES | 4397, 3783, 3542, 2991, 1903, 1778, 1418 |
| miR-144 | NO |  | YES | No target given |
| miR-146a | YES | 1858 | YES | No target given |
| miR-181b | NO |  | YES | No target given |
| miR-23b-5p | NO |  | NO |  |
| miR-147 | NO |  | YES | 3994, 3314 |
| miR-1224 | YES | No target given | NO |  |
| miR-154a | NO |  | NO |  |
| miR-31 | YES | 7116, 4448, 2807, 1454, 513 | YES | No target given |
| miR-150 | YES | No target given | YES | 8027, 1770, 258 |

1. **Vejnar CE, Zdobnov EM.** 2012. MiRmap: comprehensive prediction of microRNA target repression strength. Nucleic Acids Res **40:**11673-11683.

2. **Hsu PW, Lin LZ, Hsu SD, Hsu JB, Huang HD.** 2007. ViTa: prediction of host microRNAs targets on viruses. Nucleic Acids Res **35:**D381-385.
